# Cod-Liver Oil and Chemistry

**Published:** 1895-12

**Authors:** 


COD-LIVER OIL AND CHEMISTRY. By F. PECKEL MÖLLER, Ph. D. London: Peter Möller, 43 Snow Hill, E. C. New York: Wm. H. Schieffelin & Co. 1895. Printed for private distribution.

The first part of this work gives a brief account of Norway, its people and its industrial conditions. It then treats of cod-liver oil, the methods of catching the fish, the preparation of the oil and an account of its constituents. In the old days the oil was prepared by the putrefaction of the livers of the cod. Peter Möller, in 1853, was the first to render the oil from the fresh livers by means of steam. Even this product was not entirely satisfactory, as it produced disagreeable after-effects. In consequence of this, P. M. Hyerdahl began an investigation for the purpose of remedying this defect. Mr. Hyerdahl finds that the disagreeable after-effects are caused by the presence of hydroxy acids in the oil, and that these may be excluded by rendering the livers in an atmosphere of carbon dioxide, which process is being used by Peter Möller. The book gives a very readable and interesting account of cod-liver oil and its preparation.

The second part of the book is a veritable pictorial organic chemistry. It is not a work which can be used by beginners, but chemists may read it with interest and profit. J. A. M.

